# Laws Regulating the Licensing of Physicians and the Practice of Medicine

**Published:** 1888-02

**Authors:** 


### Editorial.

### LAWS REGULATING THE LICENSING OF PHYSICIANS AND THE PRACTICE OF MEDICINE.

The address of Dr. J. D. S. Davis, of Birmingham, Alabama, published in this number of THE JOURNAL, will recall to mind the deplorably chaotic condition of legislation on this subject throughout the United States, and perhaps inspire the hope of amelioration in coming years.

The first difficulty, apparently insuperable, which confronts us is the fact that each State has unrestricted authority to control the subject within its own limits, and most of them have attempted to do so by widely variant methods, so that practically there are more than thirty different systems of laws of force in the country, intended to regulate this important public interest. Without some methcd can be devised to give to them wider scope and uniformity, the wisest enactments will be in the future, as they have been in the past, of but little or temporary benefit.

Uniformity and permanency of good medical laws are so essential to their faithful execution, and are so important to the common weal as reasonably to commend them to the attention of the Federal government; but the limitation of its powers is so restricted as to preclude all hope of action on its part. The separate States, acting each for itself, alone have authority on this subject, and no scheme hitherto proposed has met the approval of any two of them.

It is difficult to devise, and often more difficult to procure, the enactment of a law which will operate with fairness and uniformity within the limits of a single State. Differences of opinion or interest exist between widely separated sections of the same State, between urban and rural populations, which are not easily reconciled. A proof of this is found in the speedy repeal or abandonment of many old laws on this subject, the multiplicity of existing enactments, and in the fact that none of them give entire satisfaction to the communities subject to them.

It is pleasant to know that these difficulties, however great, do not deter from further effort. The profession awaits with interest every new experiment, whether it be made in Illinois, Alabama, North Carolina or elsewhere, and information in regard to the successful working of any of these laws is always interesting.

Some of these plans seem to have failed by reason of the multiplicity of good objects towards which they were directed and the complexity of the machinery employed.

A few objects of generally recognized necessity, secured by simple methods, would, it is probable, be enough for a beginning of an organization. Other powers and duties could subsequently be added as experience might show to be necessary.

### ITEMS.

DR. JNO. THAD. JOHNSON has recently returned to Atlanta to resume the practice of medicine and surgery. He needs no introduction to the profession of this city and State at our hands. Coming to Atlanta years ago, without position or friends, by tact and industry, he rapidly rose in the profession, sharing both its honors and emoluments, and on his removal to California, three or four years ago, was enjoying the confidence of his professional brethren, and a large and lucrative practice.

DR. HUNTER P. COOPER, of this city, has been appointed surgeon of the West Point Railroad. The company is to be congratulated on securing the services of such an able man.

